# Circular RNAs in Human Cancer

**DOI:** 10.3389/fonc.2020.577118

**Published:** 2021-01-18

**Authors:** Xiong Wang, Huijun Li, Yanjun Lu, Liming Cheng

**Affiliations:** Department of Laboratory Medicine, Tongji Hospital, Tongji Medical College of Huazhong University of Science and Technology, Wuhan, China

**Keywords:** circRNA, miRNA, transcription, translation, peptide

## Abstract

Circular RNAs (circRNAs) are a class of endogenous single-stranded covalently closed RNAs, primarily produced from pre-mRNAs *via* non-canonical back-splicing. circRNAs are highly conserved, stable, and expressed in tissue- and development-specific pattern. circRNAs play essential roles in physiological process as well as cancer biology. By the advances of deep sequencing and bioinformatics, the number of circRNAs have increased explosively. circRNAs function as miRNA/protein sponge, protein scaffold, protein recruitment, enhancer of protein function, as well as templates for translation involved in the regulation of transcription/splicing, translation, protein degradation, and pri-miRNA processing in human cancers and contributed to the pathogenesis of cancer. Numerous circRNAs may function in diverse manners. In this review, we survey the current understanding of circRNA functions in human cancer including miRNA sponge, circRNA-protein interaction, and circRNA-encoded protein, and summarize available databases for circRNA annotation and functional prediction.

## Introduction

Circular RNAs (circRNAs) are a class of endogenous single-stranded covalently closed RNAs, primarily produced from pre-mRNAs *via* non-canonical back-splicing (1). circRNAs were first discovered in the 1970s, and were ignored as byproducts of RNA splicing (2, 3). In 2013, circRNAs were found to act as microRNA (miRNA) sponge (4, 5). circRNAs are highly conserved, and exhibit tissue-specific and development stage-dependent expression patterns (6). Both the expression and functions of circRNAs may be independent of their host genes (7). However, experimental functional evidence is still missing for most of the discovered circRNAs. Recently, by the advances in deep sequencing technologies and bio-informatic tools, the number of circRNAs have increased explosively.

Several types of circRNAs have been identified, including exonic circRNA (EcRNA), intronic circRNA (CiRNA), and exon-intron circRNA (EIcRNAs). Recently, tRNA intron derived circRNA (tricRNA) *via* pre-tRNA splicing has been reported (8). circRNAs can bind to primary miRNAs (pri-miRNA) or mature miRNA, coding region and 3’-untranslated region (3’-UTR) of mRNA directly, or recruit proteins to these regions. The multi-functions of circRNAs have been widely investigated, including miRNA or protein sponges, protein scaffold, protein recruitment, enhancer of protein function, as well as templates for translation (1). A variety of databases have been reported, including circRNA annotation, internal ribosome entry site (IRES) assessment, and open reading frame (ORF) prediction (9).

In this review, we survey the current understanding of circRNA biogenesis, miRNA sponge, circRNA-protein interaction, circRNA-encoded protein, and available databases in human cancer.

## Biogenesis of circRNAs

**Figure 1:**
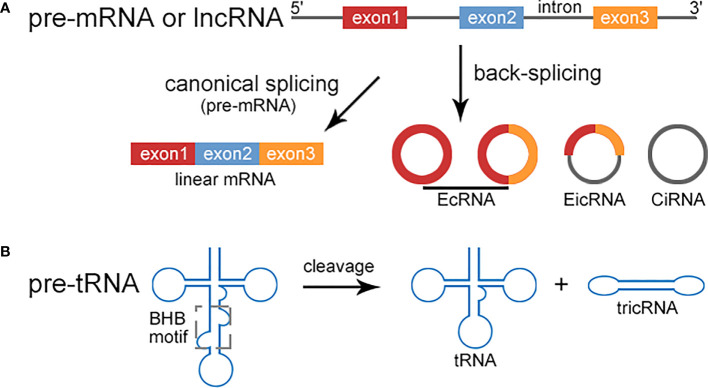
Biogenesis of circRNA. **(A)** Pre-mRNA can be transcribed into linear mRNA *via* canonical splicing or produce circRNAs including EcRNA, EIcRNA, and CiRNA through back-splicing. EcRNAs and CiRNAs only contain exons and introns respectively, while EIcRNAs consist of both. circRNAs can also be originated from lncRNAs *via* back-splicing, such as CDR1as and circLINC-PINT. **(B)** The intron excision of pre-tRNA occurs when the tRNA splicing endonuclease complex (TSEN) binds to the bulge-helix-bulge (BHB) motif to release tRNA and tricRNA.

circRNAs are usually generated from non-canonical back-splicing of pre-mRNA, ranging from hundreds to thousands of nucleotides in length. circRNAs are resistant to exonucleases (RNase R), therefore highly stable. circRNAs can be classified into three types: EcRNAs, CiRNAs, and EIcRNAs. Besides of pre-mRNAs, recent studies have revealed that circRNAs may be derived from long noncoding RNAs (lncRNAs) (**Figure 1A**). LINC00632 was found to be the host gene for CDR1as (10). circLNC-PINT was originated from the lncRNA LNC-PINT (11). In addition, tricRNAs are a novel type of circRNAs generated during pre-tRNA splicing (**Figure 1B**). A number of proteins are involved in the regulation of circRNA biogenesis, including muscleblind protein (MBL), ADAR family of RNA binding proteins (RBPs), quaking (QKI) (12).

## Mechanisms of Actions of circRNAs in Human Cancer

miRNA sponge is the most widely investigated function of circRNAs. Besides, circRNAs may function as protein sponges, protein scaffold, protein recruitment, enhancer of protein function, as well as templates for translation. Moreover, circRNAs may contribute to cancer progression in more than one way (13, 14).

### circRNAs Act as miRNA Sponges in Human Cancer

**Figure 2:**
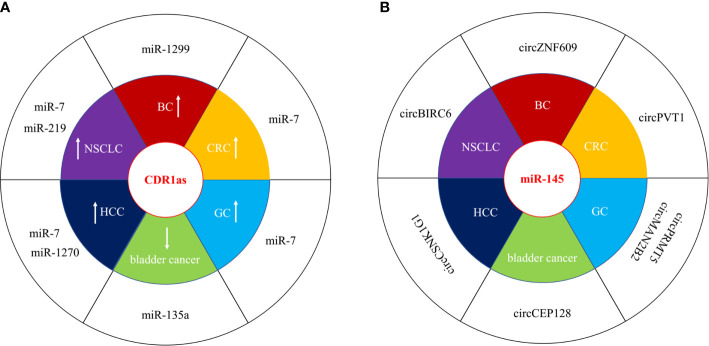
circRNAs act as miRNA sponges in human cancer. **(A)** CDR1as was both oncogenic and suppressive in different types of cancer. “↑” represented upregulated and oncogenic, “↓” represented downregulated and suppressive. **(B)** miR-145 wad sponged by several circRNAs in different types of cancer.

circRNAs can function as competing endogenous RNA (ceRNAs) or miRNA sponges to inhibit miRNA by interacting with the miRNA response elements (MREs), leading to the up-regulation of miRNA target gene. Numerous circRNAs participate to the progression of human cancer *via* acting as miRNA sponges in oncogenic or suppressive way. We have listed dys-regulated circRNAs with miRNA sponge potential in human cancer published in recent 5 years in **Table 1**, including bladder cancer, breast cancer (BC), colorectal cancer (CRC), gastric cancer (GC), hepatocellular carcinoma (HCC), and non-small-cell lung cancer (NSCLC). circRNAs show tissue specific expression and function in different human cancers. CDR1as (ciRS-7) was increased in BC, CRC, GC, HCC, and NSCLC, and acted as oncogenic, however, it was decreased and inhibited the progression of bladder cancer by sponging different target miRNAs (**Figure 2A**) (27, 29, 40, 56, 69, 87). One miRNA could be sponged by diverse circRNAs in different types of cancer. MiR-145 acted as a tumor suppressor in several cancers, and could be inhibited by a number of circRNAs, including circBIRC6, circCEP128, circMAN2B2, circPRMT5, circPVT1, circCSNK1G1, and circZNF609 (**Figure 2B**) (15, 52, 60, 65, 91, 107). The function of miRNA sponge is the most extensively studied role of circRNAs, although multi-functions of circRNAs have been identified.

**Table 1:** circRNAs act as miRNA sponges in human cancer.

Cancer	circRNA	Change	Target miRNA	Effect	Ref
Bladder cancer	circCEP128	Up	miR-145	oncogenic	(15, 16)
	circFNTA	Up	miR-370-3p, miR-451a	oncogenic	(17, 18)
	circHIPK3	Down	miR-558	suppressive	(19)
	circITCH	Down	miR-17, miR-224	suppressive	(20)
	circMTO1	Down	miR-221	suppressive	(21)
	circPTPRA	Down	miR-636	suppressive	(22)
	circVANGL1	Up	miR-605-3p, miR-1184	oncogenic	(23, 24)
	circZFR	Up	miR-377	oncogenic	(25)
	circZKSCAN1	Down	miR-1178-3p	suppressive	(26)
	CDR1as	Down	miR-135a	suppressive	(27)
	circFNDC3B	Down	miR-1178-3p	suppressive	(28)
BC	CDR1as	Up	miR-1299	oncogenic	(29)
	circABCB10	Up	miR-1271	oncogenic	(30)
	circAGFG1	Up	miR-195	oncogenic	(31)
	circANKS1B	Up	miR-148a, miR-152-3p	oncogenic	(32)
	circCDYL	Up	miR-1275	oncogenic	(33)
	circDENND4C	Up	miR-200b, miR-200c	oncogenic	(34)
	circEPSTI1	Up	miR-4753, miR-6809	oncogenic	(35)
	circGFRA1	Up	miR-34a	oncogenic	(36)
	circITCH	Down	miR-214, miR-17	suppressive	(37)
	circUBAP2	Up	miR-661	oncogenic	(38)
	circZNF609	Up	miR-145	oncogenic	(39)
CRC	CDR1as	Up	miR-7	oncogenic	(40)
	circANKS1B	Up	miR-149	oncogenic	(41)
	circCDYL	Down	miR-150	suppressive	(42)
	circCSPP1	Up	miR-361	oncogenic	(43)
	circDENND4C	Up	miR-760	oncogenic	(44)
	circHIPK3	Up	miR-7, miR-1207-5p	oncogenic	(45, 46)
	circITGA7	Down	miR-370-3p, miR-3187-3p	suppressive	(47, 48)
	circMTO1	Down	miR-19b	suppressive	(49)
	circPIP5K1A	Up	miR-1273a	oncogenic	(50)
	circPRMT5	Up	miR-377	oncogenic	(51)
	circPVT1	Up	miR-145	oncogenic	(52)
	circVAPA	Up	miR-101, miR-125a	oncogenic	(53, 54)
	circZNF609	Up	miR-150	oncogenic	(55)
GC	CDR1as	Up	miR-7	oncogenic	(56)
	circHIAT1	Down	miR-21	suppressive	(57)
	circHIPK3	Up	miR-107	oncogenic	(58)
	circLARP4	Down	miR-424	suppressive	(59)
	circMAN2B2	Up	miR-145	oncogenic	(60)
	circMTO1	Down	miR-3200-5p	suppressive	(61)
	circPIP5K1A	Up	miR-376c, miR-671	oncogenic	(62, 63)
	circPRKCI	Up	miR-545	oncogenic	(64)
	circPRMT5	Up	miR-145	oncogenic	(65)
	circPVT1	Up	miR-125	oncogenic	(66)
	circZFR	Down	miR-130a, miR-107	suppressive	(67)
	circZNF609	Up	miR-145	oncogenic	(68)
HCC	CDR1as	Up	miR-7, miR-1270	oncogenic	(69, 70)
	circABCB10	Up	miR-670-3p	oncogenic	(71)
	circBIRC6	Up	miR-3918	oncogenic	(72)
	circCDYL	Up	miR-892a, miR-328-3p	oncogenic	(73)
	circHIAT1	Down	miR-3171	suppressive	(74)
	circHIPK3	Up	miR-124	oncogenic	(75)
	circLARP4	Down	miR-761	suppressive	(76)
	circMAN2B2	Up	miR-217	oncogenic	(77)
	circMTO1	Down	miR-9	suppressive	(78)
	circMYLK	Up	miR-362-3p	oncogenic	(79)
	circPRKCI	Up	miR-545	oncogenic	(80)
	circPRMT5	Up	miR-188	oncogenic	(81)
	circPVT1	Up	miR-203, miR-3666	oncogenic	(82, 83)
	circVAPA	Up	miR-377	oncogenic	(84)
	circZFR	Up	miR-511	oncogenic	(85)
	circZNF652	Up	miR-203, miR-502	oncogenic	(86)
NSCLC	CDR1as	Up	miR-7, miR-219	oncogenic	(87–89)
	circAGFG1	Up	miR-203	oncogenic	(90)
	circBIRC6	Up	miR-145	oncogenic	(91)
	circFOXM1	Up	miR-614, miR-1304-5p	oncogenic	(92, 93)
	circGFRA1	Up	miR-183-3p	oncogenic	(94)
	circHIPK3	Up	miR-149	oncogenic	(95)
	circNT5E	Up	miR-134	oncogenic	(96)
	circPIP5K1A	Up	miR-136, miR-142-5p	oncogenic	(97, 98)
	circPRMT5	Up	miR-377, miR-382, miR-498	oncogenic	(99)
	circPTPRA	Down	miR-96	suppressive	(100)
	circPVT1	Up	miR-125b, miR-497	oncogenic	(101, 102)
	circSMARCA5	Down	miR-19b	suppressive	(103)
	circVANGL1	Up	miR-195	oncogenic	(104)
	circZFR	Up	miR-101	oncogenic	(105)
	circZKSCAN1	Up	miR-330-5p	oncogenic	(106)

CRC, colorectal cancer; HCC, hepatocellular carcinoma; NSCLC, non-small-cell lung cancer.

### circRNA-Protein Interaction in Human Cancer

circRNA-protein interaction is considered as the second-most important function of circRNAs (108). RBPs are the most well-known circRNA interacting molecules, and a number of RBPs are involved in circRNAs-mediated oncogenic or suppressive functions in human cancer. circRNAs may act as protein sponge, protein recruitment, or protein scaffolding to regulate transcription/translation directly or indirectly. Moreover, circRNA-protein interaction also contributes to the post-transcriptional regulation of target protein, including ubiquitination and phosphorylation mediated degradation. These functions are described in **Table 2**.

**Table 2:** circRNA-protein interaction in human cancer.

circRNA	Type	Cancer	Change	RBP	Location	Effect	Ref
circERBB2 (hsa_circ_0007766)	EcRNA	Gallbladder cancer	Up	–	nucleus	transcription regulation	(109)
circHuR (hsa_circ_0049027)	EcRNA	Gastric cancer	Down	CNBP	nucleus	transcription regulation	(110)
circDONSON (hsa_circ_0004339)	EcRNA	Gastric cancer	Up	–	nucleus	transcription regulation	(111)
circDNMT1 (hsa_circ_0049224)	EcRNA	Breast cancer	Up	–	nucleus	transcription regulation	(112)
circXIAP (hsa_circ_0005276)	EcRNA	Prostate cancer	Up	FUS	cytoplasm/nucleus	transcription regulation	(113)
circCUX1 (hsa_circ_0132813)	EcRNA	Neuroblastoma	Up	EWSR1	nucleus	transcription regulation	(114)
circCTNNB1 (hsa_circ_0123778)	CiRNA	Gastric cancer	Up	DDX3	nucleus	transcription regulation	(115)
circSMARCA5 (hsa_circ_0001445)	EIcRNA	Glioblastoma	Down	SRSF1	–	transcription regulation	(116)
circAGFG1	–	Cervical cancer	Up	EZH2	nucleus	transcription regulation	(117)
circRHOT1	–	HCC	Up	–	nucleus	transcription regulation	(118)
circSMURF2	–	PTC	Up	–	nucleus	transcription regulation	(119)
circCTIC1	–	Colon cancer	Up	–	nucleus	transcription regulation	(120)
circSOX4 (hsa_circ_0131457)	EcRNA	NSCLC	–	–	–	Wnt/β-catenin pathway	(121)
circZKSCAN1	EcRNA	HCC	Down	FMRP	cytoplasm	Wnt/β-catenin pathway	(122)
circE2F3 (hsa_circ_0075804)	EcRNA	Retinoblastoma	Up	HNRNPK	cytoplasm	translation regulation	(123)
circBACH1 (hsa_circ_0061395)	EcRNA	HCC	Up	HuR	cytoplasm/nucleus	translation regulation	(124)
circFOXO3 (hsa_circ_0006404)	EcRNA	Breast cancer	Down	–	–	ubiquitination	(125)
circADD3 (hsa_circ_0020007)	EcRNA	HCC	Down	EZH2	–	ubiquitination	(126)
circNOL10	EcRNA	Lung cancer	Down	–	nucleus	ubiquitination	(127)
circGSK3β (hsa_circ_0007986)	EcRNA	ESCC	Up	–	cytoplasm	phosphorylation	(128)
circPTK2 (hsa_circ_0005273)	EcRNA	CRC	Up	–	–	EMT	(129)
circSKA3	–	Breast cancer	Up	–	cytoplasm	invasion	(130)
circAGO2 (hsa_circ_0135889)	CiRNA	Gastric cancer	Up	HuR	cytoplasm/nucleus	Ago2/miRNA	(131)

PTC, papillary thyroid carcinoma; ESCC, esophageal squamous cell carcinoma. “–” means none or unavailable.

#### circRNA-Protein Interaction Regulates Transcription

EcRNAs are usually located in cytoplasm, while CiRNAs are mainly located in nucleus, and regulate Pol II transcription (132). circCTNNB1, a CiRNA derived from introns of β-catenin (CTNNB1), bound DDX3 to facilitate DDX3-mediated transactivation of transcription factor Yin Yang 1 (YY1) (115). EIcRNAs can regulate the transcription of host gene. circCUX1 interacted with EWSR1 and facilitated EWSR1-mediated MYC-associated zinc finger protein (MAZ) transactivation, resulting in transcriptional activation of its host gene *CUX1* in neuroblastoma (114).

Accumulating evidence shows that ecircRNAs may also be enriched in nucleus and regulate transcription or splicing. circERBB2, containing exons 3 to 7 of *ERBB2*, bound and promoted nucleolus localization of PA2G4, a modulator of ribosomal DNA (rDNA) transcription, to form a circERBB2-PA2G4-TIFIA complex and increase Pol I activity and rDNA transcription in gallbladder cancer (109). circDONSON interacted with SNF2L subunit of the nucleosome-remodeling factor (NURF) complex and recruited the complex to the promoter region of *SOX4* and initiates *SOX4* transcription in gastric cancer (111). circHuR, consisting of exons 3 to 5 of *HuR*, interacted with the RBP CCHC-type zinc finger nucleic acid binding protein (CNBP) and restrained its binding to *HuR* promoter, resulting in down-regulation of HuR and repression of tumor progression (110).

#### circRNA-Protein Interaction Regulates Translation

EcRNAs may be involved in the regulation of translation *via* interacting with RBPs in cytoplasm. Has_circ_0075804 enhanced E2F transcription factor 3 (*E2F3*) mRNA stability through binding with HNRNPK, and promoted the proliferation of retinoblastoma in *E2F3* dependent manner (123). circBACH1 bound HuR and induced its translocation from the nucleus to the cytoplasm, where HuR disrupted the IRES in the 5’-UTR of *p27* and inhibited p27 translation, leading to the promotion of HCC growth (124).

#### circRNA-Protein Interaction Regulates Protein Degradation

Post-transcriptional regulation of proteins, including ubiquitination and phosphorylation, are linked to protein degradation. circFOXO3 recruited both MDM2 and p53 as protein scaffolding, enhancing MDM2 mediated poly-ubiquitination and degradation of p53. The increased interaction of MDM2 and p53 released Foxo3, another target protein of MDM2, and induced apoptosis by up-regulation of the Foxo3 downstream target in breast cancer (125). circADD3 interacts with EZH2 and CDK1 to promote CDK1-mediated EZH2 ubiquitination, increasing the expression of metastasis suppressors due to EZH2 induced H3K27me3 (126).

circRNA can also prevent protein degradation by inhibiting ubiquitination or phosphorylation. circNOL10 interacted with sex comb on midleg-like 1 (SCML1) to prevent its ubiquitination, and promoted the SCML1 mediated transcriptional regulation of the HN family to inhibits lung cancer development (127). circGSK3β interacted with GSK3β to release β-catenin from GSK3β mediated phosphorylation and degradation, promoting metastasis of ESCC (128).

### circRNAs Act as Template for Translation in Human Cancer

circRNAs are considered as non-coding RNAs (ncRNAs), due to the absence of 5’ and 3’ ends. Emerging evidence shows circRNAs can encode regulatory peptides. Chen et al. firstly observed Initiation of protein synthesis in artificial constructs of circRNAs *in vitro* (133). Endogenous circRNAs can also function as translation templates (134, 135). The IRES, initiation codon AUG, and ORF have been found in numerous circRNAs, indicating their protein-coding potentials. Mechanically, IRES- and N6-methyladenosines (m6A)-mediated translation initiation contribute to circRNA translation (136). We have listed circRNAs acting as template for translation in human cancer in **Table 3**.

**Table 3:** circRNAs encoded in human cancer.

circRNA	Type	Cancer	Change	Length	ORF	Peptide	Effect	Ref
circFNDC3B (hsa_circ_0006156)	EcRNA	CRC	Down	526bp	651bp	218aa	suppressive	(137)
circFBXW7	EcRNA	Glioma	Down	620bp	552bp	185aa	suppressive	(138)
circAKT3 (hsa_circ_0017250)	EcRNA	Glioblastoma	Down	524bp	519bp	174aa	suppressive	(139)
circSHPRH (hsa_circ_0001649)	EcRNA	Glioma	Down	440bp	435bp	146aa	suppressive	(140)
circLNC-PINT	EcRNA	Glioblastoma	Down	1084bp	258bp	87aa	suppressive	(11)
circβ-catenin (hsa_circ_0004194)	EcRNA	Liver cancer	Up	1129bp	1080bp	370aa	oncogenic	(141)
circPPP1R12A (hsa_circ_0000423)	EcRNA	Colon cancer	Up	1138bp	216bp	73aa	oncogenic	(142)
circLgr4	–	CRC	Up	–	54bp	19aa	oncogenic	(143)
circGprc5a	–	Bladder cancer	Up	–	30bp	11aa	oncogenic	(144)

“–” means none or unavailable.

circFBXW7, down-regulated in both glioma and breast cancer, was able to encode a 185 amino-acid (aa) peptide named FBXW7-185aa. FBXW7-185aa competitively interacted with USP28, release c-Myc from USP28 induced de-ubiquitination, leading to c-Myc ubiquitination and degradation (138, 145). CircSHPRH, another suppressive circRNA decreased in glioma, carried a unique sequence “UGAUGA” which contained both the initiation codon “UGAUGA” and termination codon is “UGAUGA” with overlapping. circSHPRH encoded a 146aa peptide named SHPRH-146aa, which protected SHPRH from ubiquitination mediated degradation, leading to suppression of cell proliferation and tumorigenicity (140). circAKT3 was the second circRNA contained overlapped start-stop codons, which carried a unique sequence “UAAUGA” including both the initiation codon “UAAUGA” and termination codon is “UAAUGA” with overlapping. circAKT3 encoded a 174aa peptide named AKT3-174aa, which negatively modulated the PI3K/AKT signal pathway in glioblastoma by competitively interacting with phosphorylated PDK1 (139). circFNDC3B, a 526bp ecircRNA derived from exons 5 and 6 of FNDC3B, harbored a predicted ORF of 651bp and took more than one whole circle to translate a novel 218aa peptide named circFNDC3B-218aa. circFNDC3B-218aa alleviated the suppressive effect of Snail on FBP1 to inhibit EMT in colon cancer (137). These studies suggest that the ORFs in circRNAs are quite different from linear mRNAs, and may be even longer than the circRNA itself due to the circle structure.

## Roles of circRNAs in Human Cancer

circRNAs are responsible for various cellular processes including proliferation, differentiation, and apoptosis. A number of literatures have explained the role of circRNAs in cancer progression, including proliferation and apoptosis, angiogenesis, EMT and metastasis, tumor microenvironment, and drug resistance. These circRNAs act as tumor promoters or suppressors. circRNAs are highly stable in serum and exempted from endonuclease degradation, showing remarkable potential of cancer diagnostic and prognostic biomarkers.

### circRNAs and Cell Proliferation and Apoptosis in Human Cancer

CircRNA expression profiling by RNA sequencing or microarray is indispensable to explore novel oncogenic and tumor suppressive circRNAs and to elucidate the underlying mechanisms. By using circRNA microarray, Sun et al. identified that circLRIG3 (hsa_circ_0027345), derived from the back splicing of pre-LRIG3 mRNA exons 2–11, was increased in HCC. They further tested the expression of circLRIG3 in 130 pairs of HCC and para-cancerous normal tissues. circLRIG3 was remarkably upregulated in HCC patients and HCC cell lines. High expression of circLRIG3 was associated with poor prognosis in HCC patients. Overexpression of circLRIG3 increased HepG2 cell viability by promoting proliferation and reducing apoptosis, and circLRIG3 depletion led to opposite effects. The gene-set enrichment analysis (GSEA) results revealed the enrichment of STAT3 pathway after circLRIG3 overexpression. circLRIG3 facilitated EZH2-induced STAT3 methylation and phosphorylation by forming a ternary complex with EZH2 and STAT3, leading to the activation of STAT3 signaling to promote proliferation and suppress apoptosis of HCC cells (146).

### circRNAs and Angiogenesis in Human Cancer

Tumor angiogenesis is a process of new blood vessels formation derived from already existing ones and it is a critical process for tumor growth and metastasis, as the newly formed blood vessels provide oxygen, nutrition, and growth factors. Angiogenesis is a consequence of hypoxic and ischemic signals under physiological conditions, whereas, it is uncontrolled and upregulated under pathological conditions. Pathological angiogenesis is characterized by increased proliferation of the endothelial cells and atypical morphology of tumor vasculature (147). Chen et al. found that circERBIN (has_circ_0001492), formed by circularization of exons 2–4 of the ERBIN gene, was significantly increased in CRC cells. circERBIN was higher in stage III/IV tissues than in stage I/II CRC samples. Tumors formed by CRC cells stably expressing circERBIN grew larger and more quickly. The number of microvessels were greatly increased in circERBIN overexpression tumors as analyzed by staining CD31, a sensitive vascular marker to evaluate tumor angiogenesis. They further confirmed that circERBIN promoted tumor angiogenesis by increasing a cap-independent protein translation of HIF-1α through miR-125a5p/miR-138-5p/4EBP-1 signaling (148).

### circRNAs and EMT in Human Cancer

EMT is an essential prerequisite for cancer metastasis, which facilitates cancer cells to transfer from epithelial phenotype to mesenchymal traits and to enhance the mobility of cancer cells to invade and metastasize. Liu et al. screened differently expressed circRNAs in oral squamous cell carcinoma (OSCC) with circRNA microarray, and found that circIGHG (hsa_circ_0000579) was upregulated by 109 folds in OSCC with the largest number of potential miRNA targets. circIGHG was increased in both OSCC patients and cell lines. High expression of circIGHG was correlated with poor prognosis in OSCC patients. miR-142-5p/IGF2BP3 pathway was further confirmed as the downstream target of circIGHG in OSCC cell lines. circIGHG promoted the expression of several EMT markers to influence OSCC metastasis, including ZEB-2 and SNAI. circIGHG was also found to promote EMT *in vivo* through xenograft tumors. Finally, circIGHG promoted OSCC metastasis *via* inducing IGF2BP3-mediated EMT by sponging miR-142-5p (149).

### circRNAs and Tumor Microenvironment

The tumor microenvironment (TME) consists of stromal cells, extracellular matrix components, endothelial cells, vasculature, immune cells, and various signaling entities. Different cell types within the TME form organ-like structures and play specific roles during tumor metastasis, including adipocytes, endothelial cells, fibroblasts, immune cells, and neuroendocrine cells. The crosstalk between TME components and tumor cells and drives tumor metastasis. Endothelial cells are a crucial component of the TME, which participate in angiogenesis and development of tumors. Yan et al. screened differently expressed circRNAs using RNA sequencing in human umbilical vein endothelial cells (HUVECs) under an HCC microenvironment. Two of the most downregulated circRNAs were validated by qPCR, circ_4911 and circ_4302. Overexpression of the two circRNAs both arrested cells at the GO/G1 stage and suppressed the proliferation and migration of HUVECs under an HCC microenvironment (150).

### circRNAs and Drug Resistance in Human Cancer

Chemotherapy and molecular targeted drug therapy are the main treatment options for cancers, as most cancers have progressed to the middle or late stages when diagnosed (151). The most common therapeutic drugs include 5-fluorouracil, cisplatin, epidermal growth factor receptor-tyrosine kinase inhibitors (EGFR-TKIs), oxaliplatin, and sorafenib. Drug resistance occurred after long-term therapy and leads to poor prognosis (152). circRNA expression profiles analysis have identified differentially expressed circRNAs between drug resistant and drug sensitive cancer patients and cell models (153), and functional studies have further explored the underling mechanisms of circRNAs involved in drug resistance in human cancer (154). By using RNA sequencing and qPCR, Huang et al. found that circAKT3 (hsa_circ_0000199) was increased in cisplatin-resistant GC cell lines. Consistent with the cell lines results, circAKT3 was significantly higher in the cisplatin-resistant GC tissues than in the sensitive tissues. Mechanically, circAKT3 increased the expression of PIK3R1and activated the PI3K/AKT signaling pathway by sponging miR-198, to promote DNA damage repair and to suppress apoptosis of GC cells (155). On the other hand, some circRNAs may act to sensitize cancer cells to drugs. circMCTP2 was found to be decreased in both cisplatin-resistant GC tissues and cells compared to cisplatin-sensitive GC tissues and cells. Moreover, a high level of circMCTP2 was correlated with a better prognosis of GC patients. circMCTP2 inhibited proliferation and autophagy while promoting apoptosis of cisplatin-resistant GC cells *via* sponging the miR-99a-5p/MTMR3 pathway (156).

### Circulatory circRNAs in Human Cancer

Screening biomarkers in extracellular body fluids is essential in the era of precision medicine. Whole circulating transcriptome has been applied in search of cancer biomarkers. In the era of precision medicine, the main objective of medical oncology is to promote the diagnosis and treatment of cancer. Liquid biopsy” (LB), aiming to screen highly sensitive and specific biomarkers for diagnosis and prognosis by non-invasive or minimally invasive means, has attracted much attention. A recent review on circRNAs including 77 studies in several types of cancers suggested the potential role of serum circRNAs as diagnostic biomarkers for cancer. This meta-analysis found that plasma circRNAs showed higher diagnostic accuracy than tissue. Moreover, combined circRNAs panel had good diagnostic efficacy for GC (157). Exosomes are phospholipid bilayer nanovesicles with a diameter of 30–150 nm in length which contain a range of molecules, including proteins, miRNAs, and circRNAs. Exosomes secreted from cancer cells often reflect the molecular characteristics of cancer cells and affect their tumorigenic potential. The biomarkers detected within exosomes were highly correlate with the tissue analysis. Furthermore, circRNAs within exosomes are protected from being degraded during circulation due to the lipid bilayer membrane of exosomes. Exosomes are relatively more stable than serum at room temperature. These aspects may suggest exosomal circRNAs good diagnostic and prognostic biomarkers (158).

## circRNA Databases

By the advances of bioinformatics, a number of databases have been provided including comprehensive databases with circRNA annotation, primer design, IRES and ORF prediction. There are also tissue specific and cancer specific circRNA databases (159, 160). CircBase, circRNADb, and CircInteractome are the most commonly used comprehensive databases (161–163). Recently, a variety of bioinformatic tools for predicting circRNA coding potential have been reported, including IRES prediction and ORF assessment (164, 165). We have listed these circRNA databases in **Table 4**.

**Table 4:** circRNA databases.

Database	Website	Function	Ref
circBase	http://www.circbase.org/	circRNA database	(161)
circAtlas 2.0	http://circatlas.biols.ac.cn/	comprehensive database of circRNAs across species	(166)
TSCD	http://gb.whu.edu.cn/TSCD/	tissue-specific circRNA database	(159)
CSCD	http://gb.whu.edu.cn/CSCD/	cancer-specific circRNA database	(160)
deepBase v2.0	http://biocenter.sysu.edu.cn/deepBase/	circRNA database	(167)
circRNADisease	http://cgga.org.cn:9091/circRNADisease/	circRNA and disease associations database	(168)
CircBank	http://www.circbank.cn/index.html	annotation and function prediction.	(169)
CircInteractome	https://circinteractome.nia.nih.gov/	primer design and function prediction	(163)
circRNADb	http://reprod.njmu.edu.cn/cgi-bin/circrnadb/circRNADb.php	annotation and function prediction	(162)
Circ2Traits	http://gyanxet-beta.com/circdb/	annotation and function prediction	(170)
circNet	http://syslab5.nchu.edu.tw/CircNet/	circRNA-miRNA prediction	(171)
RegRNA2.0	http://regrna2.mbc.nctu.edu.tw/index.html	platform for functional RNA motif prediction	(172)
RNAstructure	http://rna.urmc.rochester.edu/RNAstructureWeb/	secondary structure and RNA-RNA binding prediction	(173)
starBase v2.0	http://starbase.sysu.edu.cn/	circRNA-RNA and circRNA-RBP interaction	(174)
catRAPID	http://s.tartaglialab.com/page/catrapid_group	circRNA-protein interaction prediction	(175)
RPISeq	http://pridb.gdcb.iastate.edu/RPISeq/	circRNA-protein interaction prediction	(176)
IRESite	http://www.iresite.org	database of experimentally verified IRES structures	(177)
IRESfinder	https://github.com/xiaofengsong/IRESfinder	IRES prediction	(178)
IRESPred	http://bioinfo.net.in/IRESPred/	IRES prediction	(164)
SORFS	http://www.sorfs.org/	ORF database	(179)
ORFfinder	https://www.ncbi.nlm.nih.gov/orffinder/	ORF prediction	–
PhyloCSF	http://compbio.mit.edu/PhyloCSF	ORF prediction	(180)
OrfPredictor	http://bioinformatics.ysu.edu/tools/OrfPredictor.html	ORF prediction	(165)
SMS : ORF Finder	http://www.bioinformatics.org/sms2/orf_find.html	ORF prediction	(181)
CPC	http://cpc.cbi.pku.edu.cn	coding-potential assessment	(182)
CPAT	http://lilab.research.bcm.edu/cpat/index.php	coding-potential assessment	(183)
CircPro	http://bis.zju.edu.cn/CircPro	coding-potential assessment	(184)
CircCode	https://github.com/PSSUN/CircCode	coding-potential assessment	(185)

## Conclusions and Perspectives

Advances of deep sequencing and algorithms result in the rapid increasement of circRNA number. Developments in molecular biology, bioinformatics and computational analysis, and proteomics have driven the function of circRNAs from miRNA sponge to circRNA-RNA/circRNA-protein interaction, and circRNA coding potential. Many traditional ideas need to be corrected. Traditionally, EcRNAs are thought to be expressed and function in cytoplasm, while EIcRNAs and CiRNAs are in nucleus. However, accumulating evidence shows that EcRNAs were also enriched in nucleus and regulate transcription or splicing in human cancer, such as circERBB2 (109), circHuR (110), circDONSON (111), circDNMT1 (112), and circXIAP (113). Moreover, CiRNA can also function in cytoplasm. CircAGO2 interacted with HuR to facilitate its shuttling from nucleus to cytoplasm, where HuR was activated and enriched on the 3’-UTR of target mRNA, preventing AGO2 binding and subsequent AGO2/miRNA-mediated gene silencing (131). Beside of miRNA, circRNAs can bind to pri-miRNA, promoter/coding region/3’-UTR of linear mRNA directly (186–189). These studies suggest the regulatory effects of circRNAs on transcription, translation, and pri-miRNA processing as important RNA-binding molecules.

There are still some issues needed to be clarified urgently. A standard nomenclature system and updated comprehensive databases for circRNAs are quite necessary due to the rapid growth in the number of circRNAs. Although circBase is the most frequently used database, its last update was in 2017. CircAtlas, recently published by Wu et al., includes 1,007,087 highly reliable circRNAs, and over 81.3% of these circRNAs have been assembled into full-length sequences (166). Currently, the nomenclature was inconsistent. circFOXM1 was found as circFOXM1, circ-FOXM1, and circ_0025033 in Pubmed. circFBXW7 has been reported to encode a novel 185aa peptide driven by IRES. Yang *et al*. found that the 185aa peptide was encoded by circFBXW7 in glioma, derived from exons 3 and 4 of *FBXW7* with a length of 620 bp (138). Ye et al. later reported that hsa_circ_0001451, derived from *FBXW7*, also encoded this peptide, while hsa_circ_0001451 was 1,227 bp in length longer than 620 bp (145). A standard nomenclature system may help circRNA research.

In the future, the mystery of circRNAs will be further uncovered in the following aspects. First, the regulation of biogenesis and degradation of circRNAs, which may be involved in the tissue specific expression of circRNAs. RBPs, host gene, and methylation have been reported to regulate the biogenesis of circRNAs (12, 127), but the detailed molecular mechanisms remain unclear. How circRNAs are degraded also remains unclear. Fischer et al. revealed a structure-mediated circRNA decay by UPF1 and G3BP1 (190). Second, the driving mechanisms translatable circRNAs and functions of their translation products. IRES-driven or m6A-mediated initiation have been found to mediate circRNA translation (191). Third, circRNA-host gene interaction. The generation of circRNAs can be independent of host linear mRNA or compete with host mRNA splicing. circRNAs regulate the expression of host gene *via* sponging miRNAs which target host genes. Pre-mRNA methylation can also modify circRNA expression. How these circRNAs interact with their host genes in human cancer deserves further investigation. Last, circRNA as biomarker for diagnosis and treatment of cancers due to its stability.

In summary, circRNAs function as miRNA/protein sponge, protein scaffold, protein recruitment, enhancer of protein function, as well as templates for translation involved in the regulation of transcription/splicing, translation, protein degradation, and pri-miRNA processing in human cancers and contributed to the pathogenesis of cancer. By the advance of deep sequencing, bioinformatic algorithms, molecular biology, as well as proteomics, the mystery of circRNAs in human cancer will be uncovered gradually.

## Author Contributions

LC and YL designed this review. XW and HL collected the related paper. XW wrote the manuscript. All authors contributed to the article and approved the submitted version.

## Funding

This work was supported by grants from the National Natural Science Foundation of China (81572071 and 81000331).

## Conflict of Interest

The authors declare that the research was conducted in the absence of any commercial or financial relationships that could be construed as a potential conflict of interest.

## Abbreviations

circRNA, circular RNA; miRNA, microRNA; EcRNA, exonic circRNA; EIcRNA, exon-intron circRNA; CiRNA, intronic circRNA; tricRNA, tRNA intron derived circRNA; pri-miRNA, primary miRNA; 3’-UTR, 3’-untranslated region; IRES, internal ribosome entry site; ORF, open reading frame; RBP, RNA binding protein; ceRNA, competing endogenous RNA; MRE, miRNA response element; BC, breast cancer; CRC, colorectal cancer; GC, gastric cancer; HCC, hepatocellular carcinoma; NSCLC, non-small-cell lung cancer; m6A, N6-methyladenosine; CSC, cancer stem cell; LSCC, laryngeal squamous cell carcinoma; PDAC, pancreatic ductal adenocarcinoma; ESCC, esophageal squamous cell carcinoma; OSCC, oral squamous cell carcinoma; EOC, epithelial ovarian cancer.
